# The DREADDful Hurdles and Opportunities of the Chronic Chemogenetic Toolbox

**DOI:** 10.3390/cells11071110

**Published:** 2022-03-25

**Authors:** Marie Claes, Lies De Groef, Lieve Moons

**Affiliations:** 1 Laboratory of Neural Circuit Development and Regeneration, Department of Biology, KU Leuven, 3000 Leuven, Belgium; marie.claes@kuleuven.be; 2 Leuven Brain Institute, KU Leuven, 3000 Leuven, Belgium; lies.degroef@kuleuven.be; 3 Laboratory of Cellular Communication and Neurodegeneration, Department of Biology, KU Leuven, 3000 Leuven, Belgium

**Keywords:** chemogenetics, designer receptor activated by designer drugs (DREADD), neuromodulation, neurostimulation

## Abstract

The chronic character of chemogenetics has been put forward as one of the assets of the technique, particularly in comparison to optogenetics. Yet, the vast majority of chemogenetic studies have focused on acute applications, while repeated, long-term neuromodulation has only been booming in the past few years. Unfortunately, together with the rising number of studies, various hurdles have also been uncovered, especially in relation to its chronic application. It becomes increasingly clear that chronic neuromodulation warrants caution and that the effects of acute neuromodulation cannot be extrapolated towards chronic experiments. Deciphering the underlying cellular and molecular causes of these discrepancies could truly unlock the chronic chemogenetic toolbox and possibly even pave the way for chemogenetics towards clinical application. Indeed, we are only scratching the surface of what is possible with chemogenetic research. For example, most investigations are concentrated on behavioral read-outs, whereas dissecting the underlying molecular signature after (chronic) neuromodulation could reveal novel insights in terms of basic neuroscience and deregulated neural circuits. In this review, we highlight the hurdles associated with the use of chemogenetic experiments, as well as the unexplored research questions for which chemogenetics offers the ideal research platform, with a particular focus on its long-term application.

## 1. Introduction

Neurobiology research has undoubtedly been revolutionized following the introduction of opto- and chemogenetics. The breakthrough of targeted neuromodulation started with the introduction of optogenetics by the Deisseroth lab in 2005 and the proclamation of this technique as the “Method of the Year” in 2010 by *Nature* [1]. Optogenetics finds itself at the intersection of various disciplines, i.e., virology, genetics, biochemistry, and biology. It combines targeted expression of a light-sensitive modulator, via viral vector or transgenic approaches, with photo stimulation—typically achieved via an optical fiber connected to an external laser—to attain targeted control of specific cellular populations in an in vivo setting. A few years later, chemogenetics has been pushed forward as an alternative technique to optogenetics, replacing optics (light sensitive modulators and light stimulation) with pharmacology (drug sensitive modulators and drug stimulation). The use of chemogenetics was spearheaded after the introduction of DREADDs (Designer Receptors Exclusively Activated by a Designer Drug) in 2007 by the Roth lab [2]. As the acronym implies, DREADD is an umbrella term encompassing a group of genetically engineered G protein-coupled receptors (GPCRs) that have an altered ligand responsiveness. DREADDs are unresponsive to their native, endogenous ligands, but are instead exclusively switched on by engineered drugs [3]. For example, the DREADD prototypes hM3Dq (stimulatory) and hM4Di (inhibitory) are no longer activated by acetylcholine, yet hijacked to respond to the drug clozapine-N-oxide (CNO) [3]. Briefly, hM3Dq activation triggers the phospholipase C cascade, causing the release of intracellular calcium and membrane depolarization. On the other hand, hM4Di inhibits the adenylyl cyclase cascade and activates inward rectifying potassium channels, leading to membrane hyperpolarization [2,4,5]. Yet, many other DREADD receptors exist, such as hM3Ds and rM3D; or KORDi, which is activated by salvinorin B instead of CNO [6,7].

As compared to optogenetics, key differences of chemogenetics include no need for specialized equipment (e.g., optic fibers and lasers), minimal invasiveness in vivo, higher spatial resolution (i.e., not confined to the illuminated area) and, last but not least, the timescale [8]. Optogenetics offers a unique temporal resolution through instant, millisecond control over neuronal activity, yet is highly transient. Chemogenetics, on the other hand, instigates gradual neuromodulation though with an extended duration of action. Depending on the assessed read-out, associated biological effects are reported to endure 6–24 h after a single drug administration [9,10,11,12]. This implicates that 2 drug applications a day are sufficient to continuously modulate cellular activation, and the chronic character of chemogenetics has been put forward as one of the assets of the technique. Especially regarding long-term experiments or in the search towards a chronic therapy, the preference of chemogenetics over optogenetics follows logically. Yet, from the very dawn of chemogenetic research, important caveats of the DREADD technology that hamper both fundamental and translational research have drawn attention. Together with the rise of publications employing long-term DREADD experiments, it becomes clear that most caveats associated with chemogenetics are even more pressing in chronic applications and call for further optimization. This lack of fundamental knowledge is not per se negative and also indicates that the full capacity of what is possible with (chronic) chemogenetics is far from begin reached. In this review, we discuss the most common hurdles and unexplored research opportunities of the chronic DREADD research toolbox.

## 2. DREADDful Hurdles in (Chronic) Chemogenetic Studies

### 2.1. The DREADD Actuator CNO

**Figure 1:**
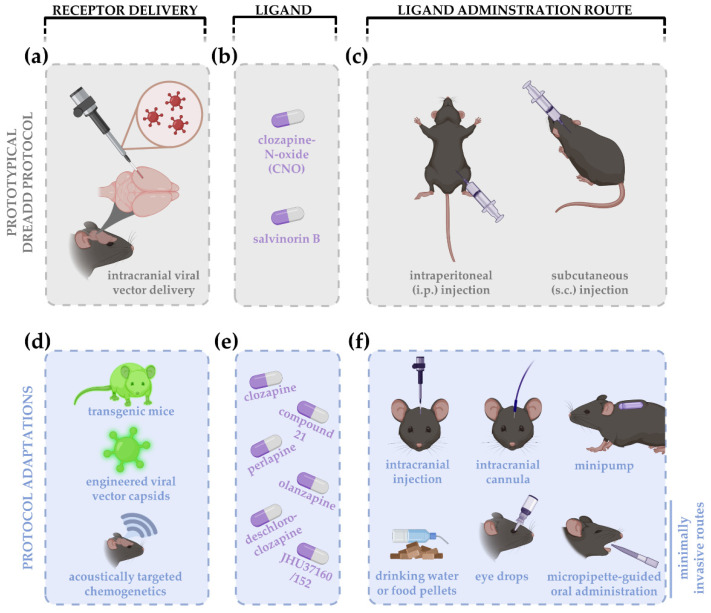
Schematic overview of a prototypical DREADD experiment (**a**–**c**) and protocol adaptations (**d**–**f**) introduced over the past years. A prototypical experiment consists of intracranial viral vector delivery of the DREADD construct (**a**). The archetypical DREADD ligand clozapine-N-oxide (CNO) (for hM3Dq, hM4Di, hM3Ds, or rM3D DREADDs), or salvinorin B (for KOR DREADDs), are typically administered via intraperitoneal (i.p.) or subcutaneous (s.c.) injections (**b**,**c**). DREADD delivery has been updated using transgenic mice and approaches to facilitate blood–brain barrier crossing, e.g., engineering of viral vector capsids or acoustically targeted chemogenetics to increase cell specificity and efficiency, respectively (**d**). Given the critiques on CNO, the use of clozapine and new generation DREADD ligands is rising, including compound 21, perlapine, deschloroclozapine, JHU37160/152, and others (**e**). Since the application of chronic chemogenetic experiments, i.p. and s.c. injections were replaced by less invasive drug administration routes, such as delivery via drinking water or food pellets, eye drops or micropipette-guided oral administration (**f**). Local administration of the DREADD ligand is often accomplished via intracranial injections, cannulas, or minipumps.

A prototypical DREADD experiment (Figure 1a–c) includes the use of the archetypal DREADD ligand CNO, yet this also represents one of the most frequently stated critiques on the platform. Evidence suggests that not CNO, but its parent metabolite clozapine, permeates the blood–brain barrier and is the actual DREADD activator in many laboratory animals, including rodents [13,14,15,16]. Clozapine is a therapeutically approved antipsychotic drug that, when present at high levels, binds to a variety of endogenous receptors with well-known effects on animal behavior [10]. Since the majority of reports employ DREADDs in behavioral studies, such off-target effects can easily confound the study results. Nonetheless, low doses of CNO (≤3 mg/kg bodyweight) are reported to result in subthreshold clozapine concentrations that are unlikely to bind with endogenous receptors, as the affinity of clozapine for DREADDs is much higher [14,17,18,19]. Although many studies demonstrated the absence of behavioral off-target effects induced by CNO or back-metabolized clozapine in animals without DREADD expression [20,21,22,23,24,25], other ligands have been developed to overcome this concern—e.g., olanzapine [26], perlapine [27], compound 21 [27], deschloroclozapine [28], and JHU37160/152 [29] (Figure 1e). The absence of off-target effects of these new generation of DREADD actuators also remains to be demonstrated. Although the number of studies including other DREADD ligands is rising, CNO is still by far the most used DREADD activator, even in chronic DREADD studies, in spite of all critiques. Whether this is related to the superiority of CNO as DREADD actuator; to its commercial availability; or to the inertia of scientific practice, i.e., the risk-averse option to stick to the most widely used and conventional method, is not clear. This led to the consensus that, regardless of the chosen DREADD actuator, findings of a DREADD study are not discounted when employing a well-considered, rigorous experimental design with proper control experiments and tailored dosing of the DREADD actuator.

One remaining question is whether clozapine should be administered as DREADD actuator instead of CNO. This could avoid variations in CNO-to-clozapine conversion, thus offering a better control of clozapine dosing [14,16]. However, in applications in which prolonged DREADD activation is required, CNO metabolism could offer some advantage as it steers a gradual production of clozapine, possibly extending the time span of neuronal manipulation [14,16]. Yet, repeated CNO administrations could also cause clozapine accumulation, reaching clozapine doses that are too high to avoid non-DREADD related side effects [30]. To draw definite conclusions, this matter should be studied in more detail. 

Although DREADDs form the leading chemogenetic platform, other approaches were also developed [6,7,31], such as the pharmacologically selective actuator/effector module (PSAM/PSEM) tool. This platform is not based on GPCR signaling, but instead hijacks ligand-gated ion channels [6,7,32]. Due to the ionotropic mechanism of action, the PSAM/PSEM platform leans more towards optogenetics, also in terms of the timescale of neuromodulation, i.e., ±30 min of neuronal activation upon stimulation [32,33]. Just as CNO, the PSEM ligand suffers from a number of flaws, primarily the need of high concentrations to achieve adequate in vivo efficiency and its short half-life [31,32,34]. Recently, so-called ultrapotent PSEM (or uPSEM in short) ligands were developed [34,35]. These ligands are highly effective at low doses and show great brain penetrance upon systemic administration in both mice and nonhuman primates, therefore showing great promise for future clinical applications. 

### 2.2. Cell Specificity

The ability to specifically modulate a single cell population whilst leaving the others unaffected offers key benefits to the neuroscience research field and can be accomplished by the DREADD platform. The most popular route to administer the DREADD ligand is via intraperitoneal/subcutaneous injection(s) (Figure 1c); yet, given the need for repeated ligand administration in chronic experiments, other systemic administration routes were introduced as well (Figure 1f). Examples include adding the designer drug to drinking water or food pellets [36,37,38], micropipette-guided oral administration [39], use of eye drops [40], or implanted minipumps [23,41]. Although one could opt for non-systemic, yet more invasive, ligand delivery routes such as the use of intracranial cannulae [42] or magnetoliposomes [43], most chemogenetic studies still apply a systemic and non-invasive administration, which implies that the DREADD construct should be specifically targeted to the cell population of interest. The DREADD construct is typically introduced via vector-mediated delivery with cell-type specific promotors, usually packed within adeno-associated viral vectors (AAVs) (Figure 1a). Upon diffusion of the vector to connected regions, off-target expression of the chemogenetic modulators can occur, which can be disadvantageous upon systemic administration of the DREADD ligand. Vector diffusion can, however, be limited by optimizing the vector’s serotype, titer, and injection volume [44]. Alternatively, to avoid off-target expression, recombinase strategies such as Cre-Lox, FLP-FRT, or Tet expression systems can be employed [9,45,46,47]. Another option to insert the DREADD modulators into the genome is via DREADD-expressing transgenic mice, with or without recombinase approaches. Currently, there are 19 chemogenetic mouse lines commercially available at the Jackson Laboratory (https://www.jax.org/research-and-faculty/tools/optogenetics-resource, accessed on 2 December 2021). Of note, the recombinase strategies can suffer from “leaky” expression, i.e., expression in the absence of the recombinase [48]. This is most certainly troublesome in transgenic mouse lines, in which the DREADD construct could have been inserted in the entire central nervous system and even in peripheral tissues, which makes it fundamentally difficult to exclude the effects of possible leaky expression on the study results. As such, localized viral vector injections still render an additional layer of specificity as compared to transgenic approaches [44].

### 2.3. Lack of Fundamental Knowledge of Chronic DREADD Neuromodulation 

Despite the fact that DREADDs were introduced more than a decade ago, their chronic use was largely unexplored until the past few years. A major advantage of chronic chemogenetic experiments is that it empowers long-term and longitudinal studies. Given the simplicity and availability of the DREADD platform, researchers adopted this plug-and-play tool in chronic experimental designs without first scrutinizing the underlying cellular and molecular actions of chronic neuromodulation. Skipping the molecular basis of chronic neuromodulation and directly probing its effect on behavioral readouts was a long shot. This is underscored by studies comparing results obtained from acute and chronic DREADD applications. Although some of those experiments show a similar level of neuronal activity or behavioral outcomes [22,49], many others report null or antagonistic effects upon continuous DREADD activation [21,22,23,36,37,38,41,50,51,52,53,54,55,56] (Table 1). Given these discrepancies, it is clear that there is no straightforward way to extrapolate study results of acute experiments to chronic experiments and more fundamental knowledge of chronic neuromodulation is of key importance. Due to the lack of research into the (molecular) basis of chronic neuromodulation via DREADDs, the exact reasons behind the diverse effects upon chronic DREADD activation remain unclear. Yet, receptor desensitization, feedback mechanisms, as well as neural plasticity have been suggested as contributing, mutually reinforcing factors and are elaborated upon in the sections below.

#### 2.3.1. Receptor Desensitization

DREADDs are hijacked GPCRs. Endogenous GPCR signaling sets off a secondary messenger chain reaction that amplifies intracellular signals and alters various physiological processes, including the membrane potential and thus neuronal (in)activation [57]. In contrast to a sole and direct altering of membrane potentials via ion channels, as achieved by the PSAM/PSEM platform, DREADD activation thus indirectly affects neuronal activation [44]. It remains unclear how chronically playing with one of the most vital signaling mechanisms of eukaryotic cells will affect the cellular and molecular physiology. On the other hand, it is well-known that overstimulation or constitutively active GPCR signaling can be destructive to the cell [58,59]. Some toxins, such as the cholera toxin, are even recognized to hijack GPCR signaling, causing deleterious permanent G protein activation [60,61]. To keep GPCR signaling within bounds, endogenous GPCRs possess a memory of previous activation. They show a strong tendency to diminish their sensitivity for receptor re-activation after prolonged activation, a phenomenon called receptor desensitization [62,63,64,65]. Furthermore, upon cumulative exposure to stimuli, GPCRs might be downregulated—i.e., internalized and degraded—thereby resulting in a reduced number of receptors on the cell membrane [59,64,65]. Apart from the activation by designer drugs, DREADDs are highly identical to endogenous GPCRs and thus likely to be subjected to receptor desensitization in chronic set-ups [66]. Evidence for receptor desensitization upon repeated DREADD activation can indeed be found in literature. For example, Goossens et al. [41] studied the effects of chronic chemogenetic inhibition of hippocampal neurons in a rat model of temporal lobe epilepsy. Seizure suppression was achieved for the first 4–5 days of treatment, yet not thereafter. The authors proposed receptor desensitization as a possible mechanism behind this tolerance effect. The occurrence of receptor desensitization was also proposed by Poyraz and colleagues [36], who tried to demonstrate this concept by looking at the effect of an additional acute CNO injection at the end of a 2-week CNO application. Indeed, the additional CNO injection did not affect the behavioral readout; yet, after a 2-day washout period, behavioral effects were reinstated upon acute CNO application. This may suggest that receptor desensitization had occurred, and receptor levels were restored after 48 h of drug abstinence. 

More evidence for the existence of receptor desensitization can be found in the employment of either stimulatory or inhibitory DREADDs. The required dose of DREADD actuator is influenced by a number of factors, including the DREADD type [19,67]. Stimulatory DREADDs have a higher efficacy in eliciting neuromodulation as compared to inhibitory DREADDs; as such, the latter require a higher dose of DREADD actuator and are thus more prone to desensitization [9,19,67]. Indeed, all studies reporting desensitization-like effects used inhibitory DREADDs, except for the recent study of Libbrecht et al. [68], who linked receptor desensitization for the first time with stimulatory DREADDs, albeit using a relatively high concentration of CNO (5 mg/kg). Nevertheless, there is ample evidence in literature that chronic chemogenetic experiments with both stimulatory and inhibitory DREADDs do not necessarily lead to desensitization [22,24,49,69,70]. This could potentially be the result of DREADD overexpression, which is in some cases even orders of magnitude higher than endogenous GPCR expression. DREADD overexpression often occurs upon vector-mediated transgene delivery and could instigate receptor reserve, thereby avoiding receptor desensitization [3]. On the other hand, DREADD overexpression is also linked with constitutive activity of the receptor [71,72]. One study reported that DREADD overexpression perturbed endogenous GPCR signaling and alterations in both ion channel activity and intracellular signaling in the absence of the DREADD ligand [71]. Various other studies examining this concept did not report constitutive DREADD activity, yet when moving to clinical applications, we should invest in studying the consequences of lifelong DREADD overexpression [72]. In summary, the occurrence of receptor desensitization again advocates for thought-out dosing and administration schemes of DREADD ligands in chronic paradigms, as well as more fundamental research into the phenomenon of receptor desensitization and overexpression.

#### 2.3.2. Neuroadaptive Changes

Plasticity is highly regulated in the adult mammalian central nervous system, for example by the excitatory–inhibitory balance upon enduring network alterations [73]. An interesting detail is that endogenous GPCRs are known to play a key role in synaptic and structural plasticity, as well as in activity-related plastic phenomena such as long-term potentiation or depression [74,75,76,77]. As such, it is not surprising that continuous neuronal stimulation/inhibition via DREADDs could be accompanied by plastic events and lead to compensatory responses, which could explain the paradoxical outcomes in acute versus chronic DREADD experiments [73]. The involvement of plasticity in DREADD activation is supported by studies that report long-lasting behavioral and physiological effects that persist over time (up to 1 month) after discontinuation of chronic CNO treatment [24,78,79]. For example, Pozhidayeva et al. [24] studied binge-like drinking behavior in mice upon chronic administration of CNO in combination with both stimulatory or inhibitory DREADDs in the nucleus accumbens. Chronic CNO application reduced alcohol consumption and this effect lasted up to 1 week after discontinuation of chronic treatment. The authors reported changes in neuronal morphology potentially induced by plastic events, as well as changes in the expression profile of plasticity-related genes. Furthermore, Salesse et al. [78] chronically inhibited dopaminergic circuits in postnatal mice using DREADDs and noted that the observed increase in locomotor activity and stereotypic behavior was still present 1 month after cessation of CNO injections. Moreover, Xie et al. [79] revealed that cardiovascular dysfunction was still present 2 to 3 days after the last CNO injection in a study in which they chronically activated glial cells in the murine sympathetic ganglia via DREADDs. Interestingly, rebound effects after cessation of chronic DREADD treatments are observed as well, again hinting towards network alterations or compensations due to prolonged treatment. For example, Desloovere et al. [10] showed a suppression of epileptic seizures in a mouse model for temporal lobe epilepsy upon chronic use of inhibitory DREADDs for 3 days. Yet, 1 day after withdrawal of clozapine injections, the fraction of time spent in seizures was significantly higher and even exceeded baseline levels. A last example of adaptive changes upon chronic chemogenetic modulation is the study of Binning et al. [52]. The authors show that repetitive stimulation of microglia for 4 consecutive days instigated microglial memory formation, thereby priming these cells for future neuroinflammatory events. Indeed, after chronic microglial activation, a decreased inflammatory response was observed upon lipopolysaccharide-induced inflammation. Hence, a deeper understanding of neuroadaptive changes in chronic DREADD applications is required. 

## 3. DREADDful Opportunities

Chronic chemogenetic applications are still in their infancy and it is thus not surprising that there are still some barriers that need to be overcome, especially given the lack of fundamental knowledge underlying chronic neuromodulation. Yet, this also implies that there are still various exciting, yet underexplored research opportunities, some of which are summed up in the sections below.

### 3.1. Cellular and Molecular Fingerprints of Neuroscience

Until now, the DREADD field has predominantly focused on yes–no questions in preclinical research (e.g., does chronic neuromodulation alleviate disease progression?) and behavioral readouts are used to answer these questions. Yet, our understanding of the cellular and molecular changes underlying these behavioral effects is still limited. We are losing out on molecular keys, not only to come up with new treatment strategies, but also in terms of fundamental neuroscience. Both opto- and chemogenetics provide exciting opportunities to unravel the cellular and molecular footprint of naive and deregulated (injured/diseased) neural circuits. For example, opto- and chemogenetics have been used to decipher the pathogenesis of Alzheimer’s disease, as reviewed by Ying and Wang [80]. Strikingly, none of those enlisted studies zoomed in on the precise molecular mechanisms that coordinate the observed functional deficits. A literature review reveals that only a handful of studies (unbiasedly) unveiled the transcriptomic/proteomic profile upon chemogenetic activation, as summarized in Table 2. These studies focused on CNO-induced DREADD modulation of neurons [24,81,82,83,84,85] or astrocytes [86,87,88], and reported corresponding molecular effects on the neurons [24,81,82,84] or astrocytes [86,87,89] themselves, and/or on neighboring endothelial cells [85] or microglia [87]. Briefly, studies depicting neuronal alterations exclusively related to DREADD activation, reported an activity-dependent upregulation of several genes in the BDNF-TrkB signaling pathway [81,82] or an upregulation of multiple immediately early genes, JUNB interaction partners and a possible involvement of PKA signaling pathway [84]. Similarly, studies focusing on the astrocytic alterations described altered signaling pathways associated with neuroinflammatory responses [89], GPCR signaling [87], and calcium ion homeostasis [87] or biological functions related to immune responses, regulation of transcription, and translation and cell proliferation/growth [89]. Moreover, astrocytic activation also led to an upregulation of *Thbs1*, which is involved in synapse formation and function [86,89]. To conclude, chemogenetics perfectly lends itself to dissect the molecular footprint of chronically altered neural circuits, something that is currently underexplored. 

### 3.2. Untangling Network Activation

Not only our understanding of the molecular changes underlying chronic neuromodulation is lacking, but also the circuit interactions and the crosstalk between different cell types participating herein remain unknown. Opto- and chemogenetics offer clear advantages in dissecting these cellular interactions as these tools enable a selective manipulation of one cell population. It is highly interesting to map the effect of (continuously) activating/silencing of a particular cell population on the transcriptome of nearby cells. This activation/inhibition will trigger the entire network in the targeted area or even neighboring circuitries, and different cell types will co-operate to achieve a certain result [90,91,92,93,94,95]. For example, Park et al. [96] showed that optogenetic stimulation of a subset of dorsal root ganglion cells also provoked neurite outgrowth in neighboring, non-stimulated neurons in an in vitro set-up. Umpierre et al. [97] elegantly described altered calcium signaling in microglia upon chemogenetic modulation of neuronal activity. Moreover, Philtjens et al. [87] used DREADDs in astrocytes and did not only observe changes in the astrocytes, but also in neighboring microglia. Unfortunately, they could not report on the effect on nearby neurons, as glial cells were enriched and neurons depleted in their dissociation protocol. Yet, many other examples of transcriptomic changes in neurons upon applications of chemogenetics in astrocytes have been observed, all summarized by Salmina et al. [98]. One other case study by Chandrasekar et al. [99] showed that acute chemogenetic inactivation of parvalbumin interneurons in a mouse model of traumatic brain injury led to increased activity and survival of neighboring principal neurons, together with reduced astrogliosis. Geeraerts et al. [100] reported a neuroprotective effect of activation of post-synaptic neurons via optogenetics on non-stimulated pre-synaptic cells in the visual system of a mouse glaucoma model. Similarly, and also in the adult murine visual system, Varadarajan et al. [101] showed stimulation of the regenerative capacity of non-stimulated pre-synaptic retinal ganglion cells upon activation of their target cells via DREADDs after a distal axon injury. Hence, these studies clearly reveal that modulation of one cell population, clearly affects others in their proximity. It is indeed generally accepted that neuronal activation will influence glial cells and vice versa, as they are highly entwined [102,103,104]. 

When observing an effect after neuronal activation/inhibition, one of the questions that could pop up is: “which cell type mostly affects the observed study results, the stimulated/inhibited neurons or the concomitant glial response?” DREADDs offer a powerful tool to unravel the reciprocal communication within the neuron–glial unit. Although DREADD research started with neuronal modulation and is still largely neuron-centric, researchers are extending this toolbox towards glial cells. As such, (single-cell) RNA sequencing upon (acute/chronic) DREADD modulation in different cell types could advance the field significantly. 

### 3.3. Exploring the Road towards Clinical Translation

Various preclinical chemogenetic studies reveal encouraging results in which the chronic use of DREADDs was proven as or more beneficial than acute treatment [22,38,54,55,56] (Table 2). For example, Cheng et al. [22] showed that chronic—but not acute—activation of DREADDs in cholinergic interneurons of the nucleus accumbens reversed social avoidance in a mouse model of depression. Likewise, chronic—but not acute—chemogenetic stimulation of neurons in the entorhinal cortex circuitry led to antidepressive-like effects in stressed mice [54]. Jaiswal et al. [55] reported that chronic activation of sensory/motor neurons resulted in enhanced axonal regeneration upon peripheral nerve injury in mice, as compared to acute CNO treatment. Another example is the study by Urban et al. [38], who studied the effect of acute and chronic activation of serotonergic neurons in the murine dorsal raphe nucleus. They observed antidepressant-like effects in both CNO regimes, yet a reduction in anxiety-like behavior was solely observed upon chronic activation of the serotonergic system. Similarly, Bązyk et al. [56] showed that acute as well as chronic DREADD stimulation in an amyotrophic lateral sclerosis mouse model restored synaptic impairment, though chronic DREADD stimulation resulted in more robust effects as compared to acute treatment. These results reveal that chronic chemogenetic neuromodulation might hold potential for clinical applications. 

Therapeutic application of the chemogenetic platform in patients requires gene therapy to introduce the chemogenetic modulators. Over the past few decades, numerous gene therapy applications were evaluated in clinical trials, as reviewed by Ginn et al. [105]. Some of those, mostly AAV-based, are currently approved and marketed [106]. However, there are still some concerns regarding long-term safety and efficacy of viral vectors in humans, especially with regard to their immunogenicity and oncogenic capacity [107,108]. Nevertheless, the field is advancing with cautious optimism and options for safer gene delivery are under investigation. For example, promising non-viral vector approaches for gene delivery are developed, as enumerated by Sainz-Ramos et al. [108]. Especially lipid-based nanocarriers hold great potential, which is best exemplified by the mRNA lipid vaccines against COVID-19 [108]. On the other hand, steps are also undertaken to introduce the chemogenetic modulators non-invasively via oral or intraperitoneal administration—e.g., via AAV-PHPs [109] or acoustically targeted chemogenetics [110]—and to (longitudinally) monitor the location and function of the chemogenetic modulators in vivo using positron emission tomography imaging techniques [111]. On top of gene therapy, clinical translation of the chemogenetic platform poses some additional obstacles. Not only patient-tailored dosing, but also the selection of the chemogenetic ligand—as discussed in Section 2.1—will be important considerations. New ligands are, however, being introduced at a fast pace, with each ligand alleviating the flaws of the previous one. For example, the improved uPSEMs for the PSAM/PSEM platform are synthesized from the clinically approved drug varenicline, offering positive prospects for clinical utility given its well-known pharmacology [34]. Hence, we predict that the biggest hurdle to overcome when moving forward to the translational use of chemogenetics will not be gene therapy, nor the chemogenetic ligand, but rather the consequences of DREADD overexpression and chronic neuromodulation of brain circuitries, and our limited understanding herein. Nevertheless, clinical translation of the chemogenetic platform is an exciting and possibly attainable avenue, although a long road lies ahead before the benefit–risk ratio of the chemogenetic tool is maximized and the toolbox can be moved from bench to bedside.

### 3.4. Neurotrophic Factors as an Interesting Example for a Future Therapeutic Direction

Various studies have shown a link between neuronal activation and survival/regeneration, which has been illustrated with naturally induced activity (e.g., exercise [112,113,114,115,116] or visual stimuli [117]) versus artificially induced activity (e.g., electrical [118,119,120,121] or optogenetically stimulated [96,122,123,124,125]). Similarly, increased axonal regeneration was shown in a retinal axoninjury model in the visual system of adult mice upon chemogenetic neuromodulation [101,126,127]. One possible mode-of-action behind these therapeutically beneficial effects of neuronal activation is through neurotrophic factor signaling [128,129,130,131]. An interesting molecule to focus on is brain-derived neurotrophic factor (BDNF), which is shown to be regulated in an activity-dependent manner and is associated with neuroprotection, neuroregeneration, and neuroplasticity [132,133]. Notably, although the molecular signature behind chemogenetic neuromodulation remains yet to be fully unraveled, two out of three studies that did assigned an important role to BDNF signaling (see Section 3.1) [81,82]. Moreover, a few studies also performed a targeted, biased search for the involvement of BDNF signaling. Xia et al. [134] reported that BDNF protein levels decreased upon silencing of dopaminergic neurons and, vice versa, that stimulating dopaminergic activity increased BDNF levels. Similarly, Blázquez et al. [128] confirmed increased *Bdnf* mRNA levels upon activation of dorsolateral striatal neurons in mice. Lastly, Xiu et al. [135] showed beneficial effects of BDNF supplementation in mouse models of obesity and diabetes, effects that could be mimicked with chronic neuronal activation of the dorsal raphe nucleus. As reduced concentrations of BDNF have been observed in many psychiatric and neurodegenerative diseases in both animal models and human patients, as reviewed extensively in [136,137,138,139,140], chronically enhancing BDNF signaling via DREADDs might offer some interesting prospects towards possible therapeutic applications of the chemogenetic platform.

## 4. Outlook

Despite the reported hurdles regarding the (chronic) chemogenetic toolbox, chemogenetics offers a unique research platform to advance neuroscience in countless ways. For many years, chemogenetic research was highly focused on behavioral assays, yet this tool uniquely offers a way to dissect the molecular footprint behind these functional changes. On top of that, chemogenetics could open avenues to decipher the chronic effects of manipulating entire neuron–glia networks, in terms of plasticity as well as isolating the roles of each cell type upon neuronal/glial modulation. By exploiting the use of (chronic) chemogenetics in fundamental research, DREADDs could play an even bigger role in the study of brain disorders and associated therapeutic options. As most diseases of the central nervous system are chronic diseases, they will probably require chronic network modulation, for which chemogenetics lends itself perfectly. To augment DREADDs to clinical utility, more research into receptor overexpression, desensitization, and neuroadaptive changes is warranted. To conclude, to fully exploit the myriad of possibilities of the chemogenetic toolbox, more in-depth fundamental research is essential and a thorough consideration of experimental parameters (e.g., DREADD expression, choice of ligand, and its dose and administration scheme) tailored to each research question, remains indispensable. We predict that many exciting chemogenetic studies will emerge in the following years, which will greatly enhance our understanding of the molecular footprint of our brain, including neuron–glial interactions, brain plasticity, and pathology.

## Figures and Tables

**Table 1:** Overview of DREADD studies in which diverse effects of acute versus chronic DREADD treatments have been observed.

Authors	Targeted Area	Chemogenetic Platform		Ligand Concentration	Ligand Administration	Chronic Administration Scheme	Diverse Effects on …
Zhan et al., 2013 [53]	Murine hypothalamus (arcuate nucleus)	hM3Dq	CNO	1 mg/kg	i.p. injection(s)	every 5 h for 3 consecutive days	Animal feeding behavior
Nawreen et al., 2020 [21]	Murine prefrontal cortex	hM4Di	CNO	1 mg/kg	i.p. injection(s)	Twice daily for 2 weeks	Stress coping strategies
Jiang et al., 2020 [23]	Murine hypothalamus (arcuate nucleus)	hM3Dq	CNO	1 mg/kg (i.p.) 2 mg/kg (pump)	i.p. injection (acute) osmotic micropump (s.c., chronic)	2 weeks	Blood pressure
Torre-Muruzabal et al., 2019 [50]	Rat substantia nigra	hM3Dq	CNO	1 mg/kg	i.p. injection(s)	3 weeks of daily injections (5 days/week)	Motor deficits
Soumier and Sibille, 2014 [51]	Murine prefrontal cortex	hM4Di	CNO	0.5 mg/kg	i.p. injection(s)	Twice daily for 2 weeks	Behavioral emotionality
Binning et al., 2020 [52]	Murine microglia	hM3Dq	CNO	1 mg/kg	i.p. injection(s)	4 days of daily injections	Pro-inflammatory cytokine expression
Poyraz et al., 2016 [36]	Murine striatum	hM4Di	CNO	0.25 mg/kg	drinking water	2 weeks	Motivation
Goossens et al., 2016 [41]	Rat hippocampus	hM4Di	Clozapine, olanzapine	0.4 mg/kg/day	Osmotic minipump (s.c.)	1 week	Seizure suppression
Nation et al. [37]	Murine subfornical organ	hM3Dq	CNO	3 mg/kg/day	drinking water	3 days	Salt appetite
Cheng et al., 2019 [22]	Murine nucleus accumbens	rM3D	CNO	1 mg/kg	i.p. injection(s)	2 weeks of daily injections	Social avoidance
Yun et al., 2018 [54]	Murine entorhinal cortex circuitry	hM3Dq	CNO	2 mg/kg	i.p. injection(s)	4 weeks of daily injections	Antidepressive-like effects
Jaiswal et al., 2018 [55]	Murine spinal cord	hM3Dq	CNO	1 mg/kg	i.p. injection(s)	2 weeks of injections (5 days/week)	Axonal regeneration
Urban et al., 2016 [38]	Murine dorsal raphe nucleus	hM3Dq	CNO	2 (acute) or 5 (chronic) mg/kg	i.p. injection (acute) drinking water (chronic)	3 weeks	Antidepressive-like effects
Bązyk et al., 2020 [56]	Murine spinal cord	rM3D or PSAM	CNO or PSEM^308^	5 mg/kg	s.c. injection(s)	1 week of daily injections	Synaptic impairment

**Table 2:** Overview of DREADD studies digging into the molecular signature of chemogenetic experiments. PubMed searches with keywords “chemogenetics + sequencing”, “chemogenetics + transcriptomics”, “chemogenetics + proteomics” and “chemogenetics + array”, assessed on 2 December 2021.

Authors	Targeted Area	Chemogenetic Platform		Goal	Molecular Signature
Pozhidayeva et al., 2020 [24]	Murine nucleus accumbens	hM3Dq, hM4Di	Chronic, daily CNO injections: 1 mg/kg for 4 weeks	Study binge-like drinking behavior	Transcriptome of neurons
Hallock et al., 2020 [81]	Murine medial prefrontal cortex	hM3Dq	Single injection of 5 mg/kg CNO	Study the link of the hippocampal-prelimbic circuitry on context-fear memory retrieval	Transcriptome of neurons
Sun et al., 2021 [82]	Murine dorsal dentate gyrus	hM3Dq	Single injection of 2 mg/kg CNO	Study the suppression of antianxiety-like behavior and neurogenesis	Transcriptome of neurons
Nagai et al., 2019 [86].	Murine striatum	hM4Di	Single injection of 1 mg/kg CNO	Study the roles of neuron-astrocyte interactions in the striatum	Transcriptome of astrocytes
Philtjens et al., 2021 [87]	Murine hippocampus and cortex	hM3Dq	Chronic CNO administration via drinking water: 5 mg/kg/day for 8 weeks	Study the effect of chronic activation of astrocytes and the microglial crosstalk	Single-cell transcriptome of astrocytes and microglia
Wang et al., 2021 [83]	Rat superior cervical ganglion	hM4Di	Chronic, daily CNO injections: 3.3 mg/kg for 30 days	Study circadian disruption and remodeling after myocardial infarction	Transcriptome of neurons
Yu et al., 2020 [89]	Murine striatum	rM3Ds, hM3Dq, hM4Di	Single injection or 1 injection every other day for 5–6 weeks of 1 mg/kg CNO	Study the astrocytic response in the striatum to different experimental perturbations and their role in Huntington’s Disease	Transcriptome of astrocytes
Dumrongprechachan et al., 2021 [84]	Murine striatum	hM3Dq	Single injection of 3 mg/kg CNO	Study the proteomic landscape of the striatum	Proteome of neurons
Pulido et al., 2020 [85]	Murine cortex/hippocampus	hM3Dq, hM4Di	Single injection of 0.5 mg/kg (hM3Dq) or 1 mg/kg (hM4Di) CNO	Study how neuronal activity regulations endothelial cells in the brain	Transcriptome of endothelial cells

## Data Availability

Not applicable.
